# Clinical implications of heat shock protein 70 in oral carcinogenesis and prediction of progression and recurrence in oral squamous cell carcinoma patients: a retrospective clinicopathological study

**DOI:** 10.1186/s40001-023-01433-8

**Published:** 2023-10-27

**Authors:** Heba A. Elhendawy

**Affiliations:** Faculty of Dentistry, Mansoura University, Mansoura, Egypt

**Keywords:** Oral squamous cell carcinoma, Heat shock protein 70, Immunohistochemistry, Prognostic indicators, Recurrence, DFS

## Abstract

**Background:**

Oral cancer is a common cause of death worldwide. The search for novel biomarkers for oral cancer is an ongoing struggle. Prognostic biomarkers are of great importance in diagnosis, and prediction of the cancer outcome. There are several disagreements in oral cancer studies over the role of heat shock proteins as prognostic markers. The current study investigated HSP70 expression in diverse tissues ranging from normal oral mucosa to dysplastic oral epithelium and oral squamous cell carcinoma to determine its role in oral carcinogenesis. Moreover, HSP70 was evaluated concerning different prognostic parameters to determine its capability in predicting cancer progression. Recurrence of tumor was recorded, and patients` disease-free survival was calculated and analyzed considering HSP70 expression to determine the potential utility of HSP70 immuno-expression in predicting recurrence.

**Methods:**

A retrospective study was accomplished on 50 cases of OSCC. Biopsies from the cancerous tissue, the free surgical margin, and the normal oral mucosa were used. The grading of dysplastic epithelium and OSCCs followed the criteria of WHO classification (2017). The clinicopathological and follow-up records for each patient were retrieved. Pearson’s Chi-square test, one-way ANOVA, and post hoc tests were used to analyze the variance of HSP70 immuno-expression concerning different parameters. The Kaplan–Meier method was used to compute and visualize disease-free survival, and the log-rank test was used to analyze the data. With Cox regression, univariate and multivariate survival analyses were run. A P-value of 0.05 or less was regarded as statistically significant.

**Results:**

A significant increased expression of HSP70 was observed as the tissue progressed from normal to dysplastic epithelium, and carcinoma (*P* = 0.000). HSP70 revealed a significant increased expression by progression from mild to severe dysplasia (*P* = 0.023), and also from well to moderately and poorly differentiated carcinoma (*P* = 0.000). High HSP70 immuno-expression was significantly associated with progression of OSCC; large-sized tumors (*P* = 0.002), advanced TNM clinical stages (*P* = 0.001), positive nodal involvement (*P* = 0.001), presence of recurrence (*P* = .008), and reduced DFS (*P* = 0.014).

**Conclusion:**

HSP70 has a crucial contribution to oral carcinogenesis, and its immune-expression could potentially be used as predictor of progression and recurrence of OSCC patients.

*Trial registration:* Retrospectively registered.

## Introduction

Oral cancer makes up to approximately 4–5% of all cancers globally. Oral cancer has a high incidence rate due to various risk factors, including tobacco and alcohol use, immune defects, genetic factors, and viruses [1]. Oral cancer is considered a common cause of death with an incidence rate of about 400,000 cases, and 223,000 deaths annually worldwide [2, 3]. Death is usually observed in advanced stage of the disease [4]. About 90% of oral neoplasms and 38% of head and neck tumors are oral squamous cell carcinomas (OSCC) [5]. Approximately 4500 persons are diagnosed with oral cancer each year in Egypt, where OSCC accounts for almost 90% of all oral malignancies [6].

Regardless of the great advances in diagnosis and management, the 5-year survival rate of OSCC patients is about 50% even following successful treatment intervention. long-term prognosis is compromised by the late initial presentation of advanced tumors, tumor metastasis, and subsequent recurrence [7, 8]. Several factors have been reported for predicting the prognosis of OSCC, including clinicopathological features, e.g., clinical stage, tumor differentiation, tumor size, and treatment types, etc. [9–11]. Tumor size, cervical lymph node metastases, and recurrence are the main clinical prognostic factors [12–14].

One of the most prevalent and evolutionary highly conserved intracellular compounds is heat shock protein (HSP) [15–18]. Both prokaryotes and eukaryotes contain several copies of the HSP70 protein. Thirteen HSP70 homologs have been identified in human cells, including the cytosol, nucleus, lysosomes, extracellular space, and mitochondria, pointing to unique and organelle-specific biological functions [18]. Under physiological settings, this class of proteins maintains the vital cellular processes and shields cells against a variety of challenges, including ischemia, infections, inflammation, heavy metals, and hyperthermia [19]. Long lines of experimental data point to HSP70’s critical function in cancer [20]. It frequently acts as a biomarker for bad prognosis and is highly expressed in malignant tumors [21, 22]. HSPs were associated with the risk, progression, and prognosis of oral cancer. However, no consistent conclusion could be acquired from the literature because of the contradictory results. Additionally, there were great conflicts in the findings concerning the expression of HSP70 in cancers. Many studies concluded a significant association between increased expression and poor prognosis in cancer patients, while others presented contradictory results. For that reasons, the current study was conducted to elucidate the role of HSP70 in oral carcinogenesis by investigating its expression in different tissue levels ranging from normal oral mucosa to dysplastic oral epithelium and OSCC. Moreover, HSP70 immuno-expression was evaluated concerning different prognostic parameters to determine its capability in predicting cancer progression. Recurrence of the tumor was recorded, and patients` disease-free survival was calculated and analyzed considering HSP70 expression to determine the potential utility of HSP70 immuno-expression in predicting recurrence.

## Materials and methods

### Patients’ selection and data retrieval

In a retrospective study a total of 73 patients were included; however, only formalin fixed paraffin embedded tissue blocks from 50 patients could be retrieved and thereby included in the study. The inclusion criteria of the selected cases were as follows: 1. All patients had a confirmed diagnosis of primary OSCC according to WHO classification; 2. availability of operable formalin fixed paraffin embedded tissue blocks for all the included cases; 3. patients were treated surgically and received radiotherapy or concurrent chemo-radiotherapy, or chemotherapy after surgery; 4. none of the selected cases had received any form of adjuvant therapy prior to their surgery; 5. all the enrolled cases were aroused from the oral cavity; 6. completion of at least 3 years follow-up; 6. complete clinical, treatment, and follow-up records for all cases. Exclusion of cases was made on the basis of having insufficient or inoperable biopsy specimen, missing of medical, clinical, and follow-up records, death due to OSCC non-related events. Biopsies from the cancerous tissue along with the free surgical margin on the periphery of the tumors were included in the study. Twenty tissue blocks of the free surgical margin group were examined and categorized into mild, moderate, and sever epithelial dysplasia. Also, the enrolled OSCC had been graded following the criteria of WHO classification into well, moderately, and poorly differentiated carcinomas [23]. Five specimens of the normal oral mucosa were used as a control group. The studied cases were chosen from the Pathology lab and Oncology unit archives at Mansoura University, Faculty of Medicine's Oncology Centre.

Clinicopathological, medical, and follow-up records were obtained for all patients. Following the completion of the treatment, patients were followed (every 3 months) with clinical examinations, abdomen ultrasonography, chest X-rays, bone scans, and ultrasonography when relapse was suspected. The medical reports included information about the disease-free survival (DFS) over the 3 years that follow the treatment. DFS was calculated from the dates of diagnosis to death, relapse, or the last follow-up. The present study was approved by IRB and the national research ethics committee in accordance with the 1964 Helsinki Declaration and its later amendments (Faculty of Dentistry ethical committee, Mansoura University IRB). Informed consent was obtained from all participants involved in the study.

### Immunohistochemistry

The paraffin blocks of OSCC tissue were divided into 4-micron-thick sections. Slides with coatings were used for tissue sections. Deparaffinization is followed by rehydration using different concentrations of alcohol and water. Antigen retrieval was carried out using a 0.01 M citric acid buffer (pH = 6.0) heated in the microwave for ten minutes. Following a 15-min incubation in methanol that contained 3% H2O2 to inactivate endogenous peroxidase, slices were then washed with distilled water. Using a rabbit polyclonal antibody against humans and a primary antibody to HSP70 (rabbit monoclonal antibody, Catalogue: 4873; Cell Signaling, Danvers, MA, USA), the slides were incubated at 4 ℃ for an overnight period. The tissue sections were examined in a semi-quantitative manner, assessing both staining intensity and percentage of positive cells as previously described in the literature [24–26]. Immunoreaction was carried out using the streptavidin–biotin complex method and overnight incubation. Four grades (intensity scores) of staining intensity for HSP70 were assigned: 0 (negative, no staining), 1 (weak, light brown), 2 (moderate, brown), and 3 (strong, dark brown). Five grades (percentage scores) were assigned to the positive percentage: 0 (0%), 1 (1–25%), 2 (26–50%), 3 (51–75%), and 4 (76–100%). The formula used to calculate staining positivity was overall scores = percentage score multiplied by intensity score. The staining scores’ results were used to get the final staining score for HSP70 (0–12). The ideal cutoff score was 4, with values over 4 indicating high expression of HSP70 and scores below 4 indicating low expression.

### Statistical analysis

One-way ANOVA and post hoc tests were used to analyze the data to compare the variations in HSP70 expression between the three groups (OSCC, dysplastic oral epithelium, and normal oral mucosa). HSP70 expression was evaluated concerning various clinicopathological factors. Additionally, the Chi-square test was applied to the data. For each inquiry, two-sided *P*-values were provided in detail. The Kaplan–Meier method was used to compute and visualize DFS, and the log-rank test was used to analyze the data. The Cox regression model was used to conduct both univariate and multivariate survival analyses to identify the independent prognostic factor. A *P*-value of 0.05 or less was regarded as statistically significant. The Statistical Package for Social Science (SPSS) version (22) and Excel program were used to statistically analyze the data.

## Results

The current retrospective study was carried out on 50 cases of OSCC. Biopsies from the cancerous tissue along with the free surgical margin on the periphery of the tumors were included in the study. Five specimens of the normal oral mucosa were used as a control group.

### Clinicopathological characteristics of the considered cases

About two-thirds of the considered cases were males (31 cases, 62%), while the rest of the cases were females (19 cases, 38%). Patient ages ranged from 29 to 80 years old with a range of 51 years, and mean 59.24 ± 10. Table 1 presents the frequencies and HSP70 expression considering the different clinicopathological parameters.

**Table 1:** The Frequencies and HSP70 expression considering the different clinicopathological parameters

Clinicopathological characteristics	Groups	HSP70 expression			Frequency	Sig./test
		Negative	low	High		
Gender	Male	0	9 (29%)	22 (71%)	31 (62%)	0.041/X^2^
	Female	0	1 (5.3%)	18 (94.7%)	19 (38%)	
Age groups	≤ 55 years	0	4 (25%)	12 (75%)	16 (32%)	0.544/X^2^
	> 55 years	0	6 (17.6%)	28 (82.4%)	34 (68%)	
Tumor site	Tongue	0	2 (11.8%)	15 (88.2%)	17 (34%)	0.280/ANOVA
	Lips and cheeks	0	3 (30%)	7 (70%)	10 (20%)	
	Floor of the mouth	0	0	8 (100%)	8 (16%)	
	Palate	0	2 (40%)	3 (60%)	5 (10%)	
	Alveolar ridge	0	3 (30%)	7 (70%)	10 (20%)	
TNM stage	Stage I and II	0	10 (38.5%)	16 (61.5%)	26 (52%)	0.001/X^2^
	Stage III and IV	0	0	24 (100%)	24 (48%)	
Tumor size	T1 and T2	0	10 (35.7%)	18 (64.3%)	28 (56%)	0.002/X^2^
	T3 and T4	0	0	22 (100%)	22 (44%)	
Nodal involvement	Negative	0	10 (38.5%)	16 (61.5%)	26 (52%)	0.001/X^2^
	Positive	0	0	24 (100%)	24 (48%)	
Distant metastasis	Absent	0	10 (25%)	30 (75%)	40 (80%)	0.077/X^2^
	Present	0	0	10 (100%)	10 (20%)	
Incidence of recurrence	Absent	0	10 (31.3%)	22 (68.8%)	32 (64%)	0.008/X^2^
	Present	0	0	18 (100%)	18 (36%)	
The studied groups	Normal oral mucosa	5 (100%)	0	0	5 (6.7%)	0.000/ANOVA
	Free surgical margin	0	14 (70%)	6 (30%)	20 (26.7%)	
	OSCC	0	10 (20%)	40 (80%)	50 (66.7%)	
Free surgical margin grading	Mild dysplasia	0	9 (100%)	0 (0%)	9 (45%)	0.023/ANOVA
	Moderate dysplasia	0	3 (50%)	3 (50%)	6 (30%)	
	Sever dysplasia	0	2 (40%)	3 (60%)	5 (25%)	
Carcinoma histologic grading	Well differentiated	0	10 (62.5%)	6 (37.5%)	16 (32%)	0.000/ANOVA
	Moderately diff	0	0	20 (100%)	20 (40%)	
	Poorly diff	0	0	14 (100%)	14 (28%)	

### HSP70 immunohistochemical expression concerning the different clinicopathological variables

HSP70 protein was located in the cell cytoplasm (mainly) and nucleus. HSP70 presented differential expression in different tissues. Negative HSP70 expression was noted in all the worked cases of normal oral mucosa (5 cases, 100%), while the free surgical margin epithelium presented mainly low (14 cases, 70%) expression in contrast with the carcinoma group that presented mainly high expression (40 cases, 80%).

Regarding the clinical variables; females presented predominantly high HSP70 expression (18 cases, 94.7%), while males reported both high (22 cases, 71%) and low (9 cases, 29%) expression. The gender of the patient had a statistically significant impact on the expression of HSP70, according to Pearson’s Chi-square test (*P* = 0.041). Three-quarters of patients who were younger than 55 years had high HSP70 expression (12 cases, 75%), while the rest revealed low expression (4 cases, 25%). Patients who were older than 55 years showed low (6 cases, 17.6%) and high (28 cases, 82.4%) expression. No statistically significant difference in HSP70 expression according to patient age was found by Pearson’s Chi-square test (*P* = 0.544).

The greater number of the studied cases presented a persistent ulcer or patch in the posterolateral surface of the tongue (17 cases, 34%), followed by lips and cheeks, and the alveolar ridge that represented by ten cases for each site (20%). Eight cases aroused from the mouth floor (16%), and five cases from the soft palate (10%). One-way ANOVA test revealed no variance in HSP70 expression among tumors of different oral sites (*P* = 0.280).

About one-half of the worked carcinomas presented a localized illness (stage I and II, 26 cases, 52%), while the other half presented advanced disease (stage III and IV, 24 cases, 48%). Carcinomas of the clinical stages I and II revealed low (10 cases, 38.5%) and high HSP70 expression (16 cases, 61.5%), while all carcinomas (24 cases, 100%) of advanced stages (III and IV) demonstrated high expression. Pearson Chi-square test revealed a high statistically significant difference in HSP70 expression concerning the TNM clinical stage (*P* = 0.001).

Comparing different sizes of tumors regarding HSP70 expression revealed; two-thirds of the small-sized tumors group (T1 and T2 tumors) showed high HSP70 immuno-expression (18 cases, 64.3%), while the rest of the cases showed low HSP70 expression (10 cases, 35.7%).

All the large-sized tumors (T3 and T4) showed high expression (22 cases, 100%). Pearson Chi-square test revealed a statistically significant difference in HSP70 expression considering the different sizes of tumors (*P* = 0.002). Moreover, the expression of HSP70 in the T1 tumors group was significantly different than the other groups (T2, T3, and T4), whereas *P* values were (0.000), while no statistically significant difference was reported among the three groups T2, T3, and T4. Table 2 presents multiple comparisons between different tumor sizes regarding HSP70 expression utilizing one-way ANOVA post hoc test.

**Table 2:** Multiple comparisons between different tumor sizes regarding HSP70 expression utilizing one-way ANOVA post hoc test

(I) Tumor size	(J) Tumor size	Mean Difference (I-J)	Std. error	Sig	95% confidence interval	
					Lower bound	Upper bound
T1	T2	− 0.818^*^	0.123	0.000	− 1.07	− 0.57
	T3	− 1.000^*^	0.130	0.000	− 1.26	− 0.74
	T4	− 1.000^*^	0.144	0.000	− 1.29	− 0.71
T2	T1	0.818^*^	0.123	0.000	0.57	1.07
	T3	− 0.182	0.091	0.052	− 0.37	0.00
	T4	− 0.182	0.110	0.106	− 0.40	0.04
T3	T1	1.000^*^	0.130	0.000	0.74	1.26
	T2	0.182	0.091	0.052	0.00	0.37
	T4	0.000	0.118	1.000	− 0.24	0.24
T4	T1	1.000^*^	0.144	0.000	0.71	1.29
	T2	0.182	0.110	0.106	− 0.04	0.40
	T3	0.000	0.118	1.000	− 0.24	0.24

^*^The mean difference is significant at the 0.05 level

Twenty-four of the analyzed cases (48%) had positive nodal metastases. Significantly, all these cases (100%) presented high HSP70 expression. The cases that were free from nodal metastasis revealed low (10 cases, 38.5%) and high (16 cases, 61.5%) HSP70 expression. Pearson Chi-square test revealed a statistically significant difference in HSP70 expression considering the status of nodal involvement (*P* = 0.001). Moreover, there was a statistically significant difference between N0 to N1 tumors (*P* = 0.004), and N0 to N2 tumors (*P*=0.004). No significant difference was reported between N1 and N2 tumor groups; they usually present similar expressions. Table 3 presents multiple comparisons between different nodal stages considering HSP70 expression utilizing one-way ANOVA post hoc test.

**Table 3:** Multiple comparisons between different nodal stages considering HSP70 expression utilizing one-way ANOVA post hoc test

(I) nodal stage	(J) nodal stage	Mean difference (I-J)	Std. error	Sig	95% confidence interval	
					Lower bound	upper bound
N0	N1	− 0.385^*^	0.126	0.004	− 0.64	− 0.13
	N2	− 0.385^*^	0.126	0.004	− 0.64	− 0.13
N1	N0	0.385^*^	0.126	0.004	0.13	0.64
	N2	0.000	0.148	1.000	− 0.30	0.30
N2	N0	0.385^*^	0.126	0.004	0.13	0.64
	N1	0.000	0.148	1.000	− 0.30	0.30

^*^The mean difference is significant at the 0.05 level

Distant metastasis was reported in 10 cases (20%). High HSP70 expression was reported in all cases that had the M1 stage, and in three-fourths of M0 cases. Pearson Chi-square test revealed no statistically significant difference in HSP70 expression regarding the incidence of distant metastasis (*P* = 0.077).

**Fig. 1:**
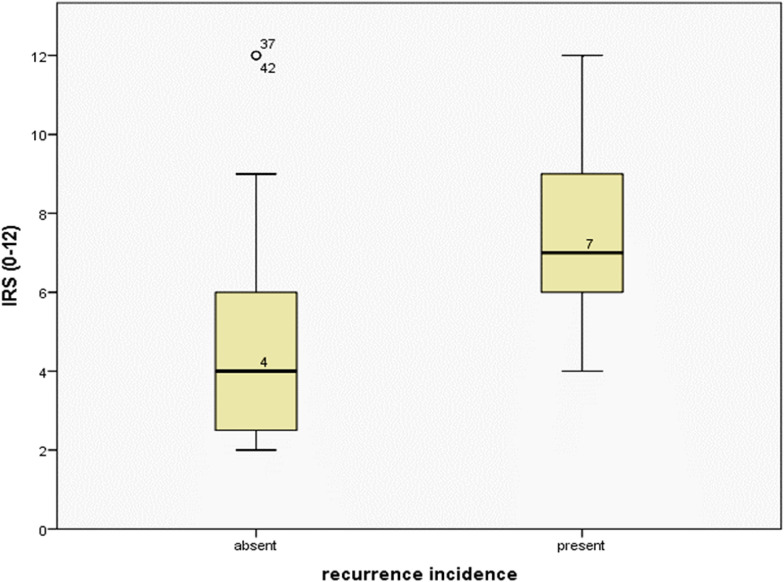
Comparison between the means of HSP70 immunoreactive score in recurrence-free and recurrence-positive cases

Recurrence was reported in 18 of the studied cases (36%), during the routine follow-up events. High HSP70 expression was significantly observed in all cases with a positive history of recurrence (18 cases, 100%), and in about two-thirds of the recurrence-free cases (22 cases, 68.8%). A high statistically significant difference in HSP70 expression was related to the incidence of recurrence using Pearson Chi-square test (*P* = 0.008, Fig. 1).

With regard to pathological variables, there was a high statistically significant difference in HSP70 expression among the worked three groups (normal oral mucosa group, the free surgical margin group, and the OSCC group, *P* = 0.000, one-way ANOVA). All the five paraffin blocks of normal oral mucosa demonstrated negative expression, while the free surgical margin group revealed (predominantly) low (14 cases, 70%), and high (6 cases, 30%) expression. OSCC group presented (predominantly) high (40 cases, 80%), and low (10 cases, 20%) expression.

**Fig. 2:**
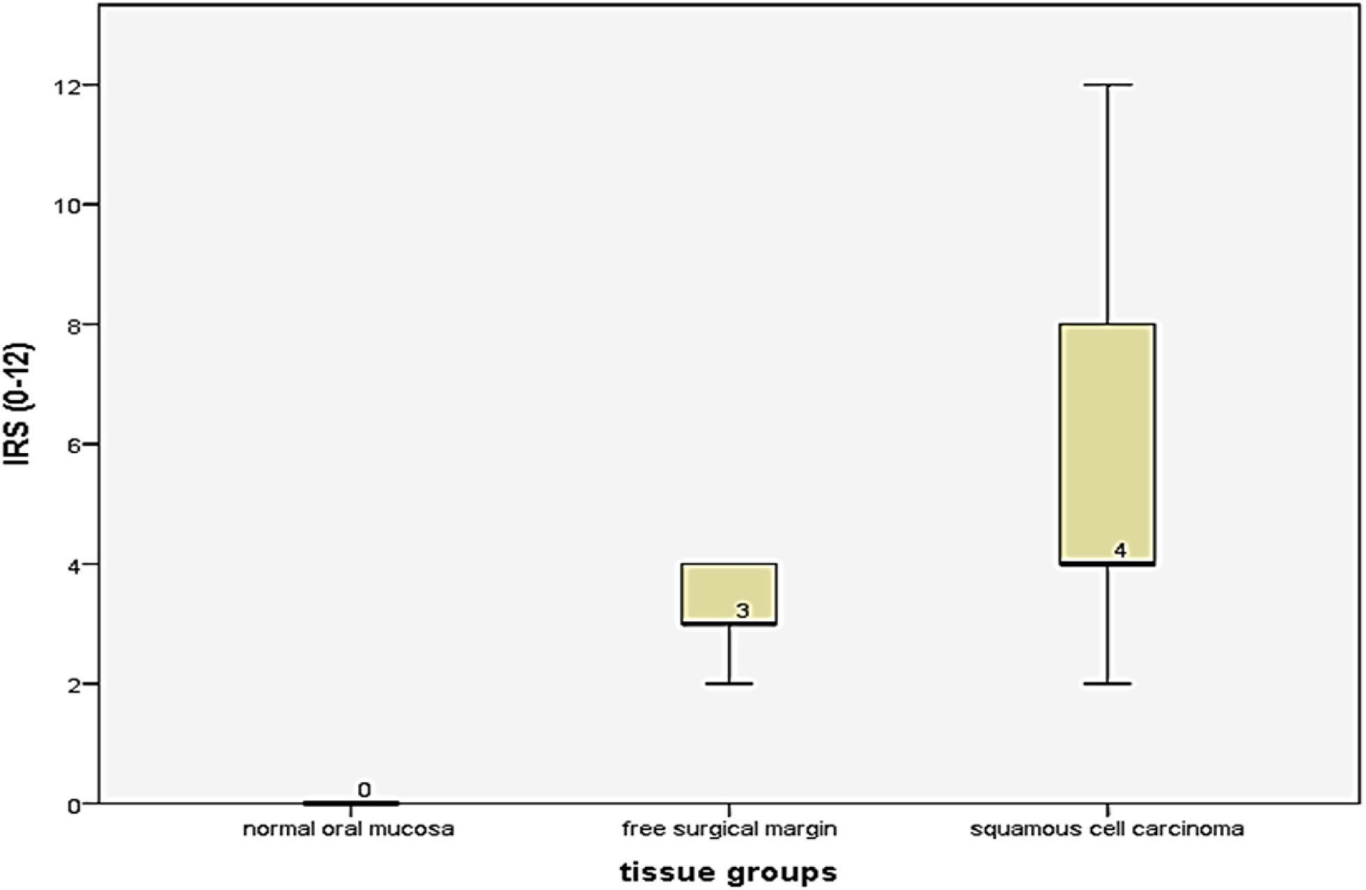
HSP70 immunoreactive scores across the worked groups of the study

HSP70 immunoreactive score (IRS) for each case was calculated by multiplying the intensity to the percentage of positivity scores. IRS ranged from 0 to 12. Normal oral mucosa group had the score 0 in all the examined cases, while the free surgical margin group got the scores 2 (4 cases, 20%), 3 (10 cases, 50%), and 4 (6 cases, 30%). Regarding the carcinoma group, the scores ranged from 2 to 12 as illustrated in Fig. 2.

**Fig. 3:**
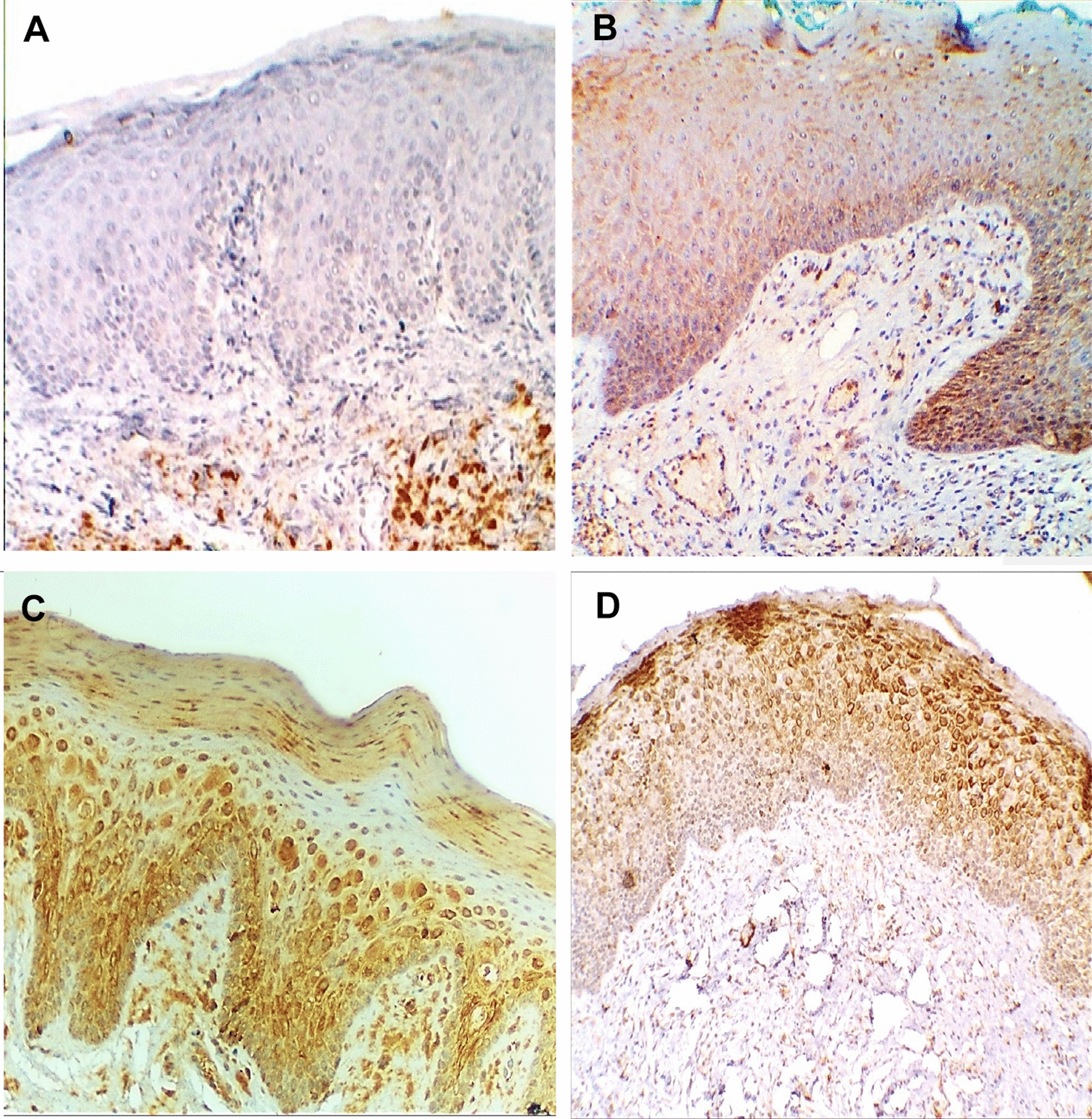
HSP70 immuno-expression in **A** normal oral mucosa, **B** mild, **C** moderate, and **D** severe epithelial dysplasia (ABC- DAB, × 250)

Moreover, HSP70 immuno-expression revealed differential expression along the three grades of epithelial dysplasia in the free surgical margin group; the mild epithelial dysplasia group demonstrated only weak expression (9 cases, 100%), while moderate and severe epithelial dysplasia groups relatively had a similar way of expression. Weak HSP70 immuno-expression was reported in three cases (50%) of the moderate dysplasia group, and two cases (40%) of the severe dysplasia group. High HSP70 expression was reported in three cases of each moderate (50%), and severe (60%) dysplasia group. One-way ANOVA post hoc test for multiple comparisons revealed a statistically significant difference in HSP70 expression between mild epithelial dysplasia group to moderate (*P* = 0.029) and severe (*P* = 0.015) dysplasia groups, while there was no statistically significant difference between moderate and severe dysplasia groups (*P* = 0.684), Table 4, Fig. 3.

**Table 4:** Multiple comparisons among three grades of dysplasia in the free surgical margin group considering HSP70 expression utilizing one-way ANOVA post hoc test

(I) Dysplasia grade	(J) Dysplasia grade	Mean difference (I-J)	Std. error	Sig.	95% confidence interval	
					Lower bound	Upper bound
Mild epithelial dysplasia	Moderate epithelial dysplasia	− 0.500^*^	0.210	0.029	− 0.94	− 0.06
	Sever epithelial dysplasia	− 0.600^*^	0.222	0.015	− 1.07	− 0.13
Moderate epithelial dysplasia	Mild epithelial dysplasia	0.500^*^	0.210	0.029	0.06	0.94
	Sever epithelial dysplasia	− 0.100	0.241	0.684	− 0.61	0.41
Sever epithelial dysplasia	Mild epithelial dysplasia	0.600^*^	0.222	0.015	0.13	1.07
	Moderate epithelial dysplasia	0.100	0.241	0.684	− 0.41	0.61

*The mean difference is significant at the 0.05 level

**Fig. 4:**
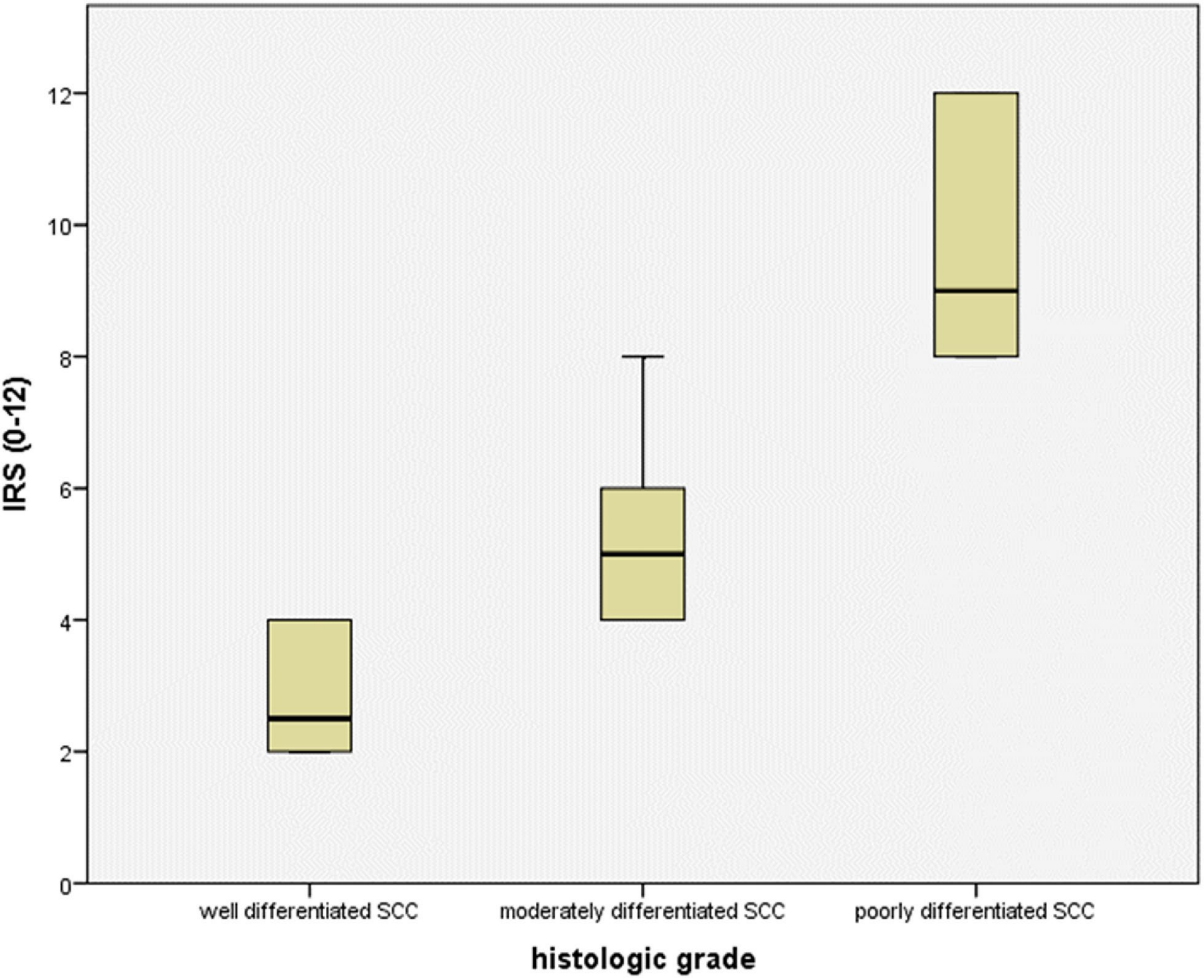
HSP70 immunoreactive scores across the different carcinoma histologic grades

**Fig. 5:**
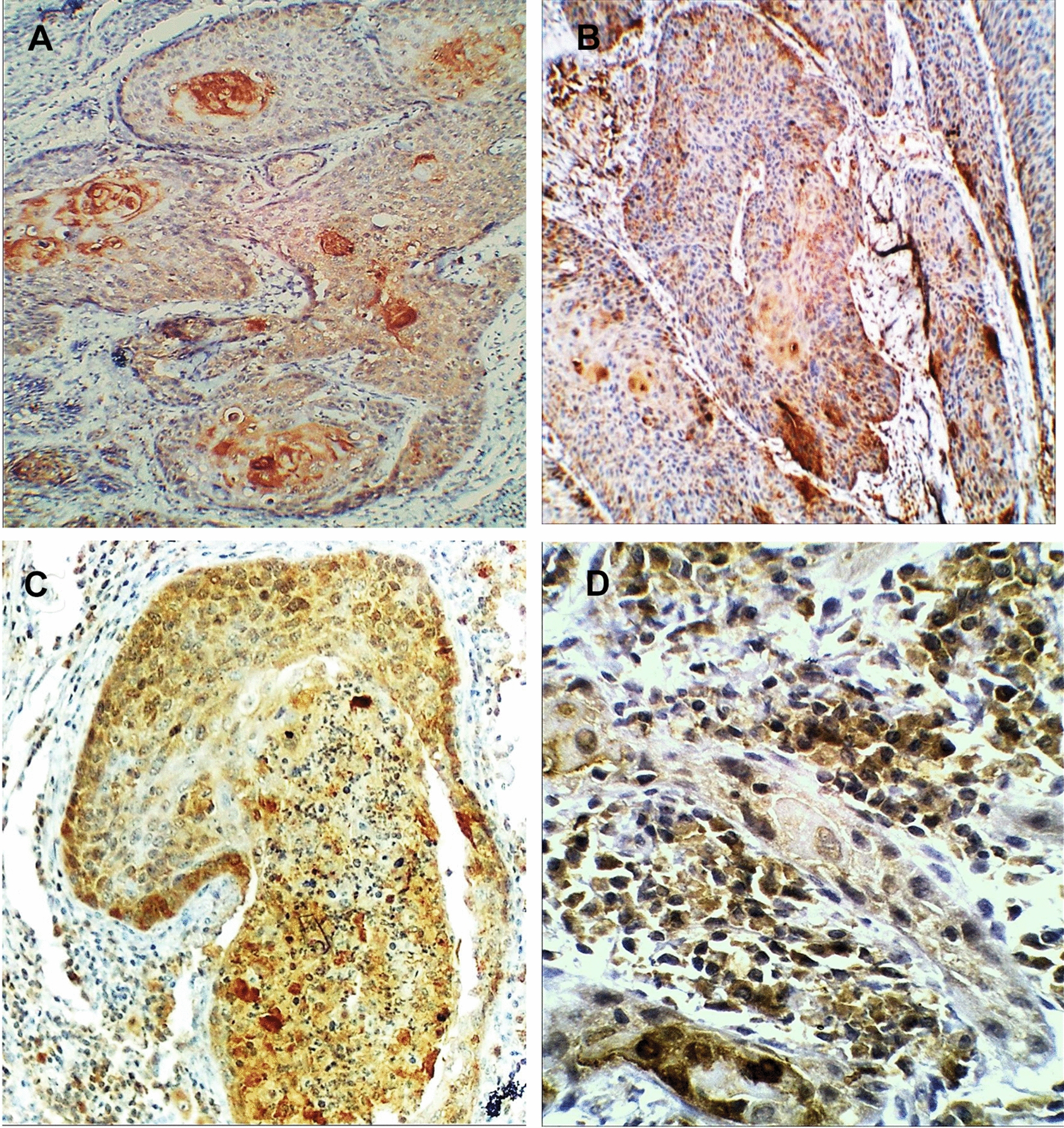
HSP 70 immuno-expression in **A**, **B** well-differentiated OSCC, **C** moderately differentiated OSCC, and **D** poorly differentiated OSCC (ABC-DAB, × 250, × 400)

Considering the varied histologic grades of OSCC, HSP70 revealed significantly high expression in moderately (20 cases, 100%) and poorly (14 cases, 100%) differentiated carcinoma groups, while well-differentiated carcinomas presented (mainly) weak (10 cases, 62.5%) and high (6 cases, 37.5%) immuno-expression. One-way ANOVA post hoc test for multiple comparisons revealed a statistically significant difference in HSP70 expression between well-differentiated carcinoma group to moderately (*P* = 0.000) and poorly (0.000) differentiated groups, while there was no statistically significant difference between moderately and poorly differentiated carcinoma groups (*P* = 1.000, Table 5, Figs. 4, 5)**.**

**Table 5:** Multiple comparisons among three histologic grades of carcinoma considering HSP70 expression utilizing one-way ANOVA post hoc test

(I) Histologic grade	(J) Histologic grade	Mean Difference (I-J)	Std. error	Sig.	95% confidence interval	
					Lower bound	Upper bound
Well differentiated SCC	Moderately differentiated SCC	− 0.625^*^	0.095	0.000	− 0.82	− 0.43
	Poorly differentiated SCC	− 0.625^*^	0.103	0.000	− 0.83	− 0.42
Moderately differentiated SCC	Well differentiated SCC	0.625^*^	0.095	0.000	0.43	0.82
	Poorly differentiated SCC	0.000	0.098	1.000	− 0.20	0.20
Poorly differentiated SCC	Well differentiated SCC	0.625^*^	0.103	0.000	0.42	0.83
	Moderately differentiated SCC	0.000	0.098	1.000	− 0.20	0.20

*The mean difference is significant at the 0.05 level

### Disease-free survival (DFS)

**Fig. 6:**
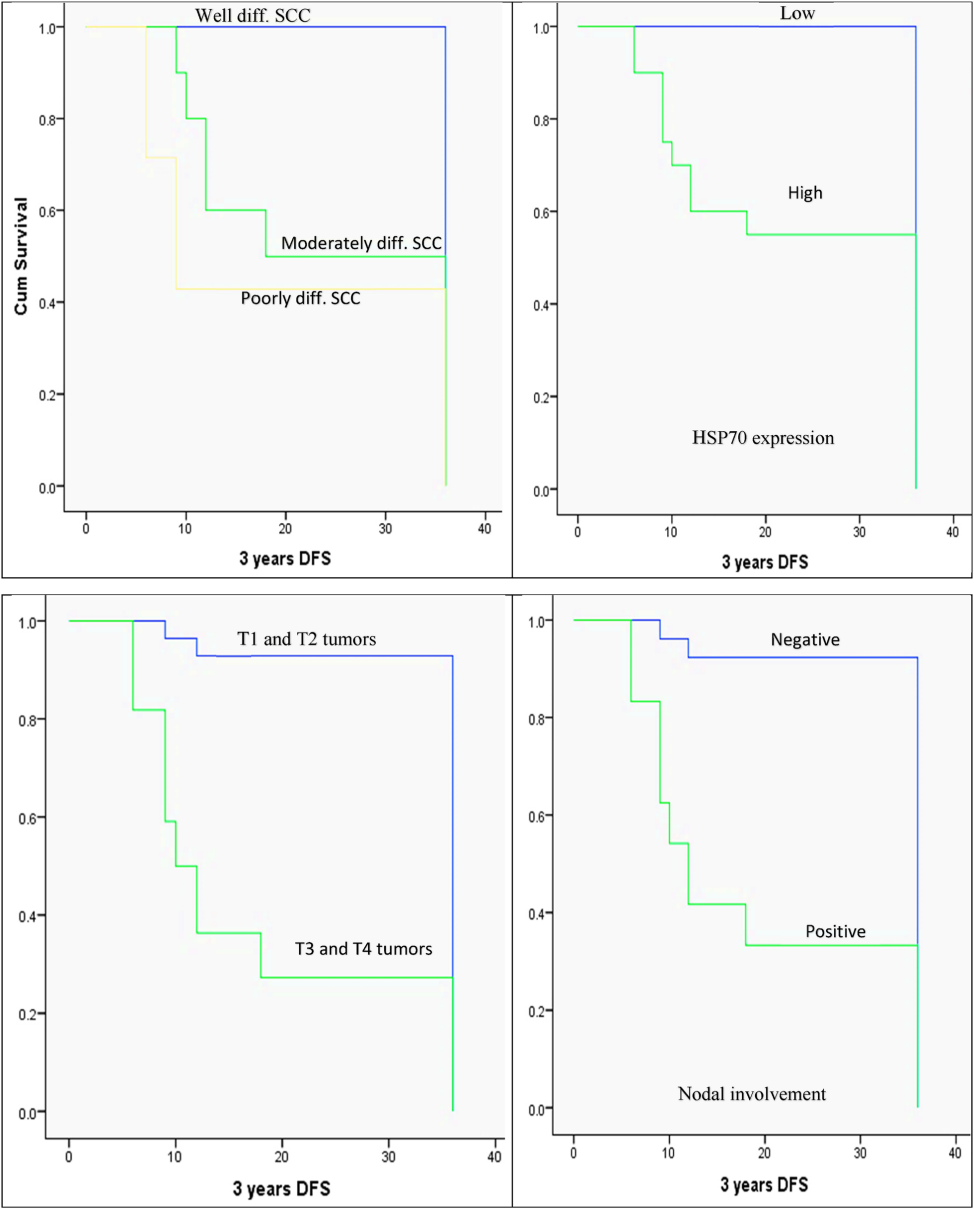

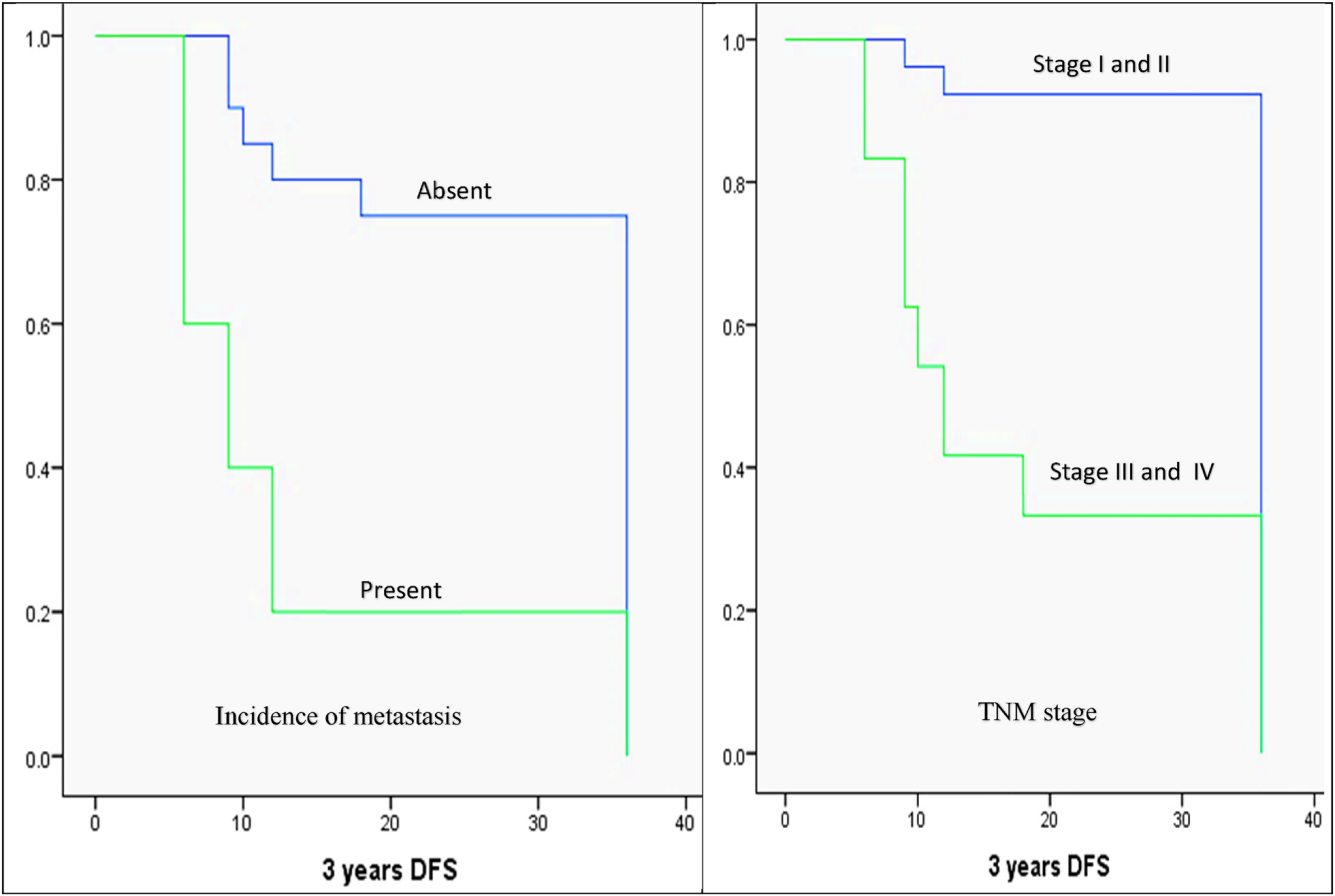
The Kaplan–Meier survival plots demonstrate the 3-year DFS that significantly reduced in moderately and poorly differentiated carcinomas, high HSP70 expression, large-sized carcinomas (T3+T4), positive nodal involvement, presence of distant metastasis, and advanced TNM stage (stage III+IV)

The Kaplan–Meier method, log-rank test, and Cox regression model were used to analyze patients’ DFS concerning the various clinicopathologic factors. Using the Kaplan–Meier method for univariate analysis, it was discovered that DFS was significantly reduced in moderately and poorly differentiated carcinomas (24.1 and 19.7 months, respectively) versus well-differentiated carcinomas (36 months, *P* = 0.001), cases with high levels of HSP70 expression (24.35 months) compared to low levels (36 months, *P* = 0.014), large-sized carcinomas (T3+T4; 17.13 months) compared to small-sized carcinomas (T1+T2; 34.17 months, *P* = 0.000), positive nodal involvement (18.7 months) compared to negative nodal involvement (34 months, *P* = 0.000), positive distant metastasis (13.8 months) compared to negative distant metastasis. On the other hand, the patient’s age (*P* = 0.338), gender (*P* = 0.310), and intraoral tumor site (*P* = 0.053) did not show a statistically significant difference in DFS (Fig. 6).

**Fig. 7:**
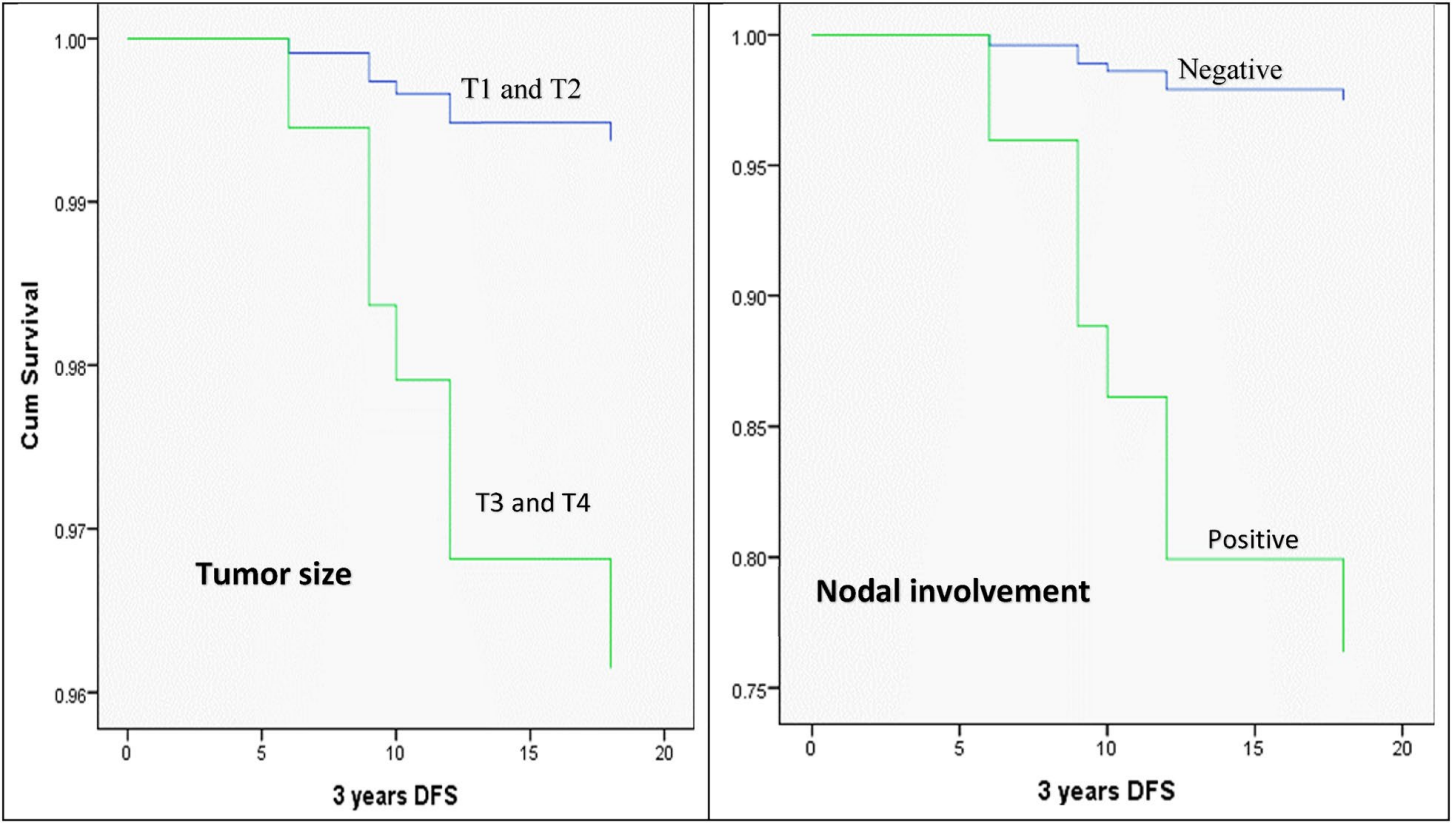
The Cox regression model illustrates tumor size and nodal involvement as the independent predictors for DFS in OSCC patients

For the purpose to learn more about whether the expression of HSP70 was an independent prognostic factor for patients with OSCC, a multivariate Cox proportional hazard regression analysis was also performed. Histological grade, nodal metastasis, tumor size, and HSP70 expression were all considered in the multivariate analysis. The findings showed that nodal involvement and tumor size are the independent predictors of patient disease-free survival (DFS) (Table 6 and Fig. 7).

**Table 6:** The Cox regression model illustrates the independent predictor(s) of DFS

Prognostic parameters	B	SE	Wald	Sig	Exp (B)	95.0% CI for Exp (B)	
						Lower	Upper
SCC grade			0.936	0.626			
SCC grade (1)	− 11.186	223.667	0.003	0.960	0.000	0.000	3.369E + 185
SCC grade (2)	− 0.464	0.480	0.933	0.334	0.629	0.246	1.611
HSP70 overall score	0.000	282.917	0.000	1.000	1.000	0.000	6.598E + 240
Tumor size	− 1.833	0.753	5.923	0.015	0.160	0.037	0.700
Nodal involvement	− 2.362	0.794	8.852	0.003	0.094	0.020	0.447

## Discussion

In numerous biological processes, such as cell differentiation, gene expression, immune system control, cellular senescence, and programmed cell death, HSPs play a crucial role [27–32]. HSPs are essential for invasiveness, cell proliferation, angiogenesis, and cancer development [33, 34]. They are involved in the development and spread of tumors [35]. Numerous physiologic and pathological processes, including immunoregulation, apoptosis, tumor treatment resistance, oncogene activation, and tumor suppressor gene inactivation have been linked to HSPs [36–41]. Numerous tumors, including colorectal, breast, prostate, lung, ovarian, gastric, oral, and esophageal malignancies, have been found to express HSPs abnormally [23, 42]. In the current study, HSP70 expression has been evaluated in a spectrum of normal, dysplastic oral epithelium, and OSCC to elucidate its contribution to oral carcinogenesis. Additionally, the expression was analyzed concerning the tumor histologic grade of differentiation, and patients` parameters like sex, tumor site, and age. Moreover, HSP70 expression was evaluated in relation to different tumor sizes, the incidence of nodal and distant metastasis, and finally the incidence of tumor recurrence.

The conducted study presented differential expression of HSP70 in different tissues (normal oral mucosa, dysplastic oral epithelium, and OSCC). Negative HSP70 expression was noted in all the worked cases of normal oral mucosa, while the dysplastic oral epithelium presented mainly low expression in contrast with the carcinoma group that presented mainly high HSP70 expression. In the same line as our work, many studies reported the same finding; Kaur et al. [43] reported increased levels of HSP70 expression in oral cancer than in potentially malignant lesions. The authors deduced the implication of HSP70 in oral carcinogenesis depending on that finding. Moreover, Markopoulos AK et al. [44] assessed the expression of HSP70 in OSCC, Leukoplakia with dysplasia, and normal oral tissue. They also documented Intensified expression of HSP70 in OSCC compared with dysplastic lesions that presented only positive expression, and normal oral mucosa that showed negative expression. They advised the utility of HSP70 as a marker for epithelial dysplasia or epithelial malignant transformation. Oral cancer risk was substantially correlated with HSP70 overexpression [45]. Furthermore, Tekkesin et al. [46] recorded increased expression of HSP70 and HSP27 during tumorigenesis in OSCC, and Thubashini et al. [47] also observed Increased HSP70 expression from oral submucous fibrosis to OSCC. In a study carried out on nasopharyngeal carcinoma (NPC), cancerous tissue showed considerably higher levels of HSP70 expression in comparison to the non-cancerous control nasopharyngeal epithelium [48]. Lung cancer studies also registered a relatively higher risk of lung cancer with increased expression of Hsp70 [49, 50]. Induction of HSP70 expression usually occurs in response to stress [43]. This fact explains the higher level of expression in dysplastic oral epithelium and oral cancer than in normal oral mucosa.

In contradiction, Sugerman et al. [51] found that HSP70 is not a definitive marker of oral malignancy in a study carried out on OSCC, dysplastic oral epithelium, and normal oral epithelium. The contradictory findings may be owing to miss understanding of the molecular mechanisms responsible for the overexpression of heat shock proteins in cancer cells as it may be tumor specific [51]. The physiopathological characteristics of the tumor microenvironment (low glucose, pH, and oxygen) are one of the processes that cause HSP induction [52]. The other mechanism is the oncoproteins that appear during carcinogenesis as mutated P53. Kaur et al. [43] proposed that p53-HSP70 complex formation may be one of the mechanisms of stabilization of p53 protein resulting in its increased levels in potentially malignant and malignant oral lesions and may be implicated in oral carcinogenesis.

HSP70 immuno-expression not only presented differential expression along the different tissue levels but also there was significant differential expression along the three histologic grades of dysplastic oral epithelium in the free surgical margin group, where HSP70 expression was significantly intensified by progression from mild to moderate and severe dysplasia. HSP70 expression is exaggerated by acquiring the malignant characteristics of cells. Moreover, high HSP70 expression was significantly observed in moderately and poorly differentiated carcinoma groups than in the well-differentiated carcinomas group that presented (mainly) weak expression. Similar conclusions were drawn from other research, including the high levels of HSP70 expression seen in moderately and poorly differentiated carcinomas [53] and elevated levels of expression linked to carcinogenesis [54, 55]. HSP70 expression and the differentiation of oral cancer yielded conflicting results as well [56, 57]. HSP70 expression did not significantly differ across the distinct histologic types of nasopharyngeal carcinoma (differentiating and undifferentiating types) [48]. It was important to note that oral cancer development can be accompanied by varying levels of HSP70 protein production, which might account for the inconsistent results.

Regarding the clinical variables; females in our study significantly presented a predominant high HSP70 expression than males (94.7% versus 71%, respectively). The gender of the patient had a statistically significant impact on the expression of HSP70, according to Pearson’s Chi-square test (*P* = 0.041). The female sex hormones may be responsible for that difference. Opposing our findings, Thubashini et al. [47] observed no association of HSP70 expression with a patient`s gender in an oral cancer study. Moreover, Feng et al. [48] reported similar findings concerning patient gender and age also in nasopharyngeal carcinoma. Furthermore, high HSP70 expression was recorded in the Japanese population among male patients over females in lung cancer studies [50, 58]. The opposing findings may be owing to factors related to special characteristics of the worked samples, or different environmental factors.

The worked study revealed no statistically significant differences in HSP70 expression neither concerning the patient`s age group (*P* = 0.544) nor the varied intraoral sites of the tumor (*P* = 0.280). Many studies reported findings on the same line as ours [48, 49, 54]. In contrast, Thubashini et al. [47] observed Increased HSP70 expression in ages older than 50 years in OSCC patients.

HSP70 expression was strongly correlated with OSCC progression and was seen in all cases with large-sized tumors (T3 and T4, *P* = 0.002, advanced TNM clinical stages (III and IV, *P* = 0.001), positive nodal involvement, and high expression (*P* = 0.002). HSP70 expression was high in all M1 stage cases, although there was no statistically significant relationship between HSP70 expression and the incidence of distant metastasis (*P* = 0.077). On the same line as our findings, HSP70’s high level of expression was significantly associated with advanced clinical stages (III and IV), the presence of nodal metastasis, but contradicts our finding regarding metastasis as high expression was associated with the presence of metastasis in nasopharyngeal carcinoma [48]. In contrast, Lee et al. [56] found that low HSP70 expression was associated with lymph node metastasis in OSCC patients.

Consistent with our findings concerning the size and the clinical stage of the tumor, non-small cell lung cancer patients with histology of adeno and squamous cell carcinoma with significant gross tumor volume had high serum Hsp70 as compared to normal healthy controls. Additionally, there was a difference in HSP70 that was noted in serum samples between patients with early-stage and advanced-stage tumors [59]. High HSP70 expression associates with advanced clinical stage in OSCC studies [53, 54]. Patients with HSP70 overexpression exhibited a more advanced stage of oral cancer, according to Zheng et al. [60]. However, according to Taghavi’s study, HSP70 overexpression was discovered to be a protective factor for the advanced stage [57].

Kaur et al. [53] worked on human oral squamous carcinoma cells (HSC-2 cell line) and deduced the implication of HSP70 in the proliferation and survival of oral tumor cells by the anti-apoptotic function of HSP70. This finding explains its increased expression in cancer cells than normal controls, in moderately and poorly differentiated carcinomas that are characterized by high proliferation capability than well-differentiated carcinomas, and in large-sized tumors than small-sized tumors. HSP70 participates in increasing the size of tumors via suppressing apoptosis pathways. Continuously under stress, such as hypoxia and nutritional depletion, tumor cells can survive but are vulnerable to apoptosis [61]. Cancers frequently contain the protein HSP70, which has been shown to block apoptosis and promote cancer growth [62]. Notably, a reduction in HSP70 level was found to cause the death of cancer cells [63]. Both intrinsic and extrinsic apoptotic pathways were shown to be affected by HSP70. By inhibiting c-Jun N-terminal kinase (JNK) and p38, HSP70 prevents apoptosis through a caspase-dependent mechanism. JNK activity is crucial for the release of cytochrome c from the mitochondria, which triggers the intrinsic apoptotic pathway [64]. The other mechanism is caspase-independent through the interaction with death receptors and the suppression of the apoptosis-inducing factor (AIF) [65]. The apoptosis-inducing factor (AIF) necessary for DNA fragmentation is inhibited by HSP70 [66]. HSP70 is a key player in necrosis and autophagy, two alternate forms of cell death to apoptosis. HSP70 was discovered to be present in the lysosomes of cancer cells, in contrast to normal cells [67]. Numerous investigations have shown that HSP70 stabilizes lysosomal membranes, preventing cancer cells from death [67–69]. Apoptozole, a HSP70 inhibitor, has demonstrated its efficacy in encouraging apoptosis in cancer cells by inducing the release of cathepsin and permeabilization of the lysosomal membrane [70].

Similar to our findings, high expression of HSP70 has been linked to lymph node metastases in breast cancer models [71]. Inactivation of HSP70 was reported to diminish tumor invasiveness and metastatic potential in breast, cervical, and bladder cancer cell lines in subsequent studies [72]. High levels of HSP70 expression shield tumor cells from anoikis and amorphosis, two cell death processes that take place when the extracellular matrix (ECM) is lost [73, 74]. HSP70 was found to have an impact on molecules related to anoikis and amorphosis, including Akt and focal adhesion kinase (FAK) [75–77]. HSP70 stabilizes the buildup of hypoxia-inducible factor-1 (HIF-1), which is implicated in tumor angiogenesis, invasion, and metastasis [78–80] and senses low oxygen levels. Extracellular HSP70 has been demonstrated by Kim and colleagues to attach to the surface of endothelial cells and trigger angiogenesis through an ERK-dependent mechanism [72]. Additionally, it has been noted that HSP70 increases IL-5-induced angiogenesis, pointing to an inflammatory role for HSP70 in encouraging angiogenesis [70]. Furthermore, numerous studies have shown that HSP70 blocks TGF-induced EMT by reducing Smad2 phosphorylation [81–83]. Furthermore, it has been demonstrated that HSP70 inhibits the formation of reactive oxygen species (ROS) and Smad3 and Smad4 phosphorylation, thereby protecting peritoneal mesothelial cells against high glucose-induced EMT [84]. These results point to a second function for HSP70 in EMT, where HSP70-peptide complexes aid tumor cells’ shift to mesenchymal phenotype. MMP-9, a processing enzyme for cleaving the CD44 receptor involved in adhesion and migration, is released as a result of extracellular HSP70 [85–87]. Additionally, Wiskott–Aldrich syndrome family member 3 (WASF3), a protein necessary for tumor migration, invasion, and metastasis, was shown to be stabilized by HSP70 and HSP90 proteins [87–89].

Utilizing the Kaplan–Meier method, log-rank test, and Cox regression model to analyze the Patients’ DFS in relation to various clinicopathologic factors. In moderately and poorly differentiated carcinomas, carcinomas with high HSP70 expression, large-sized tumors, positive nodal involvement, positive distant metastases, and advanced TNM stage, the DFS of patients was significantly reduced, according to a univariate study. However, there was no statistically significant difference between DFS and the patient’s age (*P* = 0.338), gender (*P* = 0.310), or intraoral tumor site (*P* = 0.053). Nodal involvement and tumor size were discovered to be the independent predictors of patient DFS in a multivariate study utilizing the Cox regression model. In harmony with our finding, Kaur et al. [53] reported elevated levels of HSP70 associated decreased median disease-free survival time. In other work on human oral squamous carcinoma cells (HSC-2 cell line) they concluded that HSP70 is required for the proliferation and survival of oral tumor cells [52]. Moreover, many studies reported HSP expression was inversely correlated with survival time [54, 90]. Based on the findings of non-small cell lung cancer studies, HSP70 overexpression can be used to predict survival [91].

Contrarily, numerous studies have linked lower HSP expression with a poor prognosis for oral cancer patients [52, 55, 90]. According to Lu et al. HSP70 protein overexpression significantly increased the survival rate of patients with oral cancer [45]. A small number of other investigations found no predictive association between HSP expression and patient outcome [92]. When Ito T et al. [46] examined the tongue, normal epithelium, dysplasia, and cancer, they discovered no connection between the expression of HSP27, 60, 70, 90, or p53, and survival. Positive clinical outcomes for gastric and colon cancers supported the hypothesis that natural killer cells (NK) should target mHSP70, while lower rectal and squamous cell carcinomas had a bad prognosis, according to HSP70-membrane positive phenotype (mHSP70) research [93]. The authors concluded that the variations in these malignancies' metastatic paths can account for the discrepancies in clinical outcomes [93]. HSP70 overload causes immune tolerance to tumor antigens, which aids in the progression of tumors, while the initial release of HSP70 acts as a tumor suppressant that causes the killing of membrane-positive HSP70 tumor cells by NK cells to explain conflicting findings regarding the expression of HSP in cancer.

Heat shock proteins may not currently be on the list of helpful prognostic indicators in oral cancer due to these inconsistent results.

## Conclusion

The significant differential expression of HSP70 at different tissue levels (normal, dysplastic epithelium, and OSCC) elucidates its contribution to oral carcinogenesis. Moreover, high HSP70 expression is significantly associated with poor prognostic factors. The level of HSP70 expression in cancerous tissue is predictive for the severity and progression of cancer. Therefore, HSP70 immune-expression could potentially be used as predictor of progression and recurrence of OSCC patients.

## Limitations of the study

Although immunohistochemistry offered valuable findings in measuring HSP70 expression at different tissue levels, using other techniques such as western blotting, in situ hybridization, or PCR could enrich the work. Furthermore, while the worked sample is regarded limited to some extent, it is still valid in providing useful insights. As a result, I recommend working on larger sample in the future studies to could generalize the conclusions.

## Data Availability

The data used during the current study are available from the corresponding author on a reasonable request.

## Abbreviations

OSCC: Oral squamous cell carcinoma
HSP: Heat shock protein
IHC: Immunohistochemistry
DFS: Disease-free survival
NPC: Nasopharyngeal carcinoma
JNK: C-Jun N terminal kinase
AIF: Apoptosis inducing factor
TGF: Transforming growth factor
EMT: Epithelial mesenchymal transition
